# Potential involvement of circulating exosomal miRNA-146a in disease activity and *TRAF6* gene expression in juvenile proliferative lupus nephritis

**DOI:** 10.1136/lupus-2023-001078

**Published:** 2024-02-14

**Authors:** Poorichaya Somparn, Aunaymon Srichaimongkol, Suwaphit Jungjing, Bunsita Wanthong, Saharat Nanthawong, Leelahavanichkul Asada, Marut Tangwattanachuleepron, Pornpimol Rianthavorn

**Affiliations:** 1 Center of Excellence in Systems Biology, Faculty of Medicine, Chulalongkorn University, Bangkok, Thailand; 2 Master of Science, Major in Medical Sciences, COMMON COURSE, Faculty of Medicine, Chulalongkorn University, Bangkok, Thailand; 3 Faculty of Allied Health Sciences, Burapha University, Chonburi, Thailand; 4 Center of Excellence on Translational Research in Inflammation and Immunology, Department of Microbiology, Chulalongkorn University, Bangkok, Thailand; 5 Research Unit for Sensor Innovation, Burapha University, Chonburi, Thailand; 6 Department of Pediatrics, Chulalongkorn University, Bangkok, Thailand

**Keywords:** Lupus Erythematosus, Systemic, Inflammation, Tumor Necrosis Factor Receptor-Associated Peptides and Proteins

## Abstract

**Background:**

Juvenile SLE (JSLE) is a complex autoimmune disorder that predominantly affects children and adolescents with several unique challenges, and microRNA-146a (miRNA-146a) might be an interesting anti-inflammatory molecule. Because exosomes in the blood might protect miRNAs, the association between circulating exosomal miRNA-146a and lupus proinflammatory genes, such as *IRAK1* and *TRAF6*, was studied in peripheral blood mononuclear cells from people with JSLE.

**Methods:**

Blood samples from 12 patients were collected every 3 months until 1 year with the recorded disease activity, and quantitative real-time PCR was used to determine the circulating exosomal miRNA-146a and the gene expression (*IRAK1* and *TRAF6*).

**Results:**

The mean age was 12.60±0.43 years at diagnosis and all patients had a complete response at 12 months. According to the nanoparticle tracking analysis, the abundance of exosomes was significantly lower at 3, 6 and 12 months compared with 0 months, while the level of circulating exosomal miRNA-146a was significantly higher at 12 months than at diagnosis (p<0.001). There was a negative correlation between the level of circulating exosomal miRNA-146a expression and the level of *TRAF6* mRNA (r=−0.30, p=0.049). Moreover, there were correlations between circulating exosomal miRNA-146a and disease severity such as SLE Disease Activity Index 2000 score, anti-double-stranded DNA antibody and proteinuria (urine protein–creatinine ratio), respectively. Therefore, increasing the level of circulating exosomal miRNA-146a, which might control *TRAF6* mRNA expression, could have an effect on the production of inflammatory cytokines.

**Conclusion:**

This suggests that miRNA-146a might serve as a non-invasive biomarker to evaluate the response to treatment in patients with juvenile lupus nephritis.

**WHAT IS ALREADY KNOWN ON THIS TOPIC**

- MicroRNA-146a (miRNA-146a) is known to be a susceptibility locus for SLE due to its modulation of interferon and several proinflammatory pathways in SLE including interleukin-1 receptor-associated kinase 1 (*IRAK1*) and tumour necrosis factor receptor-associated factor 6 (*TRAF6*).
- Exosomes facilitate communication between different cells and their microenvironment by transferring information, notably miRNAs, which regulate the inflammatory response in target cells.

**WHAT THIS STUDY ADDS**

- The number of exosomes was decreased after treatment at 3, 6 and 12 months.
- Circulating exosomal miRNA-146a was upregulated at 12 months after treatment and correlated with lupus disease severity (SLE Disease Activity Index 2000 score), anti-double-stranded DNA antibody concentration and proteinuria (urine protein–creatinine ratio).
- An increase in circulating exosomal miRNA-146a expression correlated with a decrease in *TRAF6* mRNA expression.

**HOW THIS STUDY MIGHT AFFECT RESEARCH, PRACTICE OR POLICY**

- The exosomal miRNA-146a might serve as a non-invasive biomarker for evaluating the response to treatment in patients with juvenile lupus nephritis.

## Introduction

SLE is distinguished by an intricate interplay among immune dysregulation, persistent inflammation and multiorgan damage^1^ that predominantly affects women during their childbearing years. However, juvenile SLE (JSLE), defined as the onset of SLE before the age of 18 years, constitutes 15–20% of SLE cases^1^ and has distinctive characteristics, including more prominence in disease severity and major organ involvement compared with adult-onset SLE.^2^ Lupus nephritis is more common in JSLE than in patients with adult-onset SLE. Notably, approximately 50–70% of patients with JSLE develop lupus nephritis, with a predominance of proliferative lesions, referred to as ‘juvenile proliferative lupus nephritis’ (JPLN).^3^

The presence of lupus nephritis can significantly affect the overall disease course and prognosis of patients with SLE. Patients with JSLE frequently necessitate elevated doses and extended duration of immunosuppressive therapy compared with those with adult-onset SLE, thereby increasing the potential for drug-related adverse effects.^4 5^ Therefore, investigating disease activity and treatment response in patients with JPLN is of paramount importance.

Despite the multifaceted pathogenesis of SLE (environmental influences, genetic predispositions and epigenetic modifications), microRNAs (miRNAs) have recently been demonstrated to be noteworthy epigenetic elements with regulated post-transcriptional inhibitory effects on gene expression.^6^ miRNAs are better able to be detected within the extracellular space and encapsulated within exosomes, which are the small extracellular vesicles secreted by various cells, including immune cells. The protection of miRNAs within exosomes confers augmented stability to these cells, maintaining the integrity of the enclosed miRNAs, and serving as conduits for their transportation to distant target cells or tissues, facilitating intercellular communication.^7^

In particular, miRNA-146a is located at chromosome 5q33.3 and is also known to be a susceptibility locus for SLE.^8^ Previous studies have demonstrated that miRNA-146a expression is lower in the serum of patients with SLE than in healthy individuals, correlating with active disease^9 10^ and revealing an association between miRNA-146a and progression of SLE. Several evidence supports the association between the dysregulation of miRNA-146a and various autoimmune diseases, including but not limited to SLE,^10^ rheumatoid arthritis,^11^ autoimmune thyroid disorder,^12^ Graves’ disease^13^ and several other autoimmune conditions.^8^ A pilot study performed by Zhou *et al*^14^ revealed that interleukin-1 receptor-associated kinase 1 (*IRAK1*) and tumour necrosis factor receptor-associated factor 6 (*TRAF6*) are potential targets of miRNA-146a. *IRAK1* undergoes phosphorylation and associates with *TRAF6*. Subsequently, this complex triggers the activation of inhibitor of nuclear factor (NF)-κB kinase, leading to the activation and translocation of the NF-κB transcription factor into the nucleus. Activated NF-κB, in turn, stimulates the synthesis and release of inflammatory cytokines.

Motivated by these observations, the primary aim of the present study is to examine the correlation between the levels of circulating exosomal miRNA-146a and its consequential impact on downstream targets, including *IRAK1* and *TRAF6*, within the context of JPLN.

## Methods

### Patients

A longitudinal study enrolled 12 sequential patients younger than 18 years with the diagnosis of JPLN from 2020 to 2022. All participants fulfilled the 2019 European Alliance of Associations for Rheumatology/American College of Rheumatology classification criteria for SLE.^15^ The study participants underwent kidney biopsy at the time of diagnosis of lupus nephritis (month 0). The indications for kidney biopsy in children with SLE at our institution included (1) urine protein-to-creatinine ratio exceeding 0.5 mg/mg or persistently active urine sediment, defined as more than 10 red blood cells/high-power field or cellular casts; and (2) renal function impairment defined as a glomerular filtration rate (GFR) <90 mL/min/1.73 m^2^. Kidney biopsy results were interpreted based on the 2003 International Society for Nephrology/Renal Pathology Society.^16^ The activity and chronicity indices were derived from composite semiquantitative scores according to the previously described protocol.^17^

Complete remission was determined using the criteria of the Kidney Disease: Improving Global Outcomes Glomerulonephritis Work Group.^18^ The criteria for complete remission were a urine protein-to-urine creatinine ratio <0.5 mg/mg and a GFR ≥90 mL/min/1.73 m^2^.

Patient characteristics, treatment regimens and serum samples were collected at diagnosis (month 0), and at 3, 6 and 12 months after diagnosis. Disease activity was determined by the SLE Disease Activity Index 2000 (SLEDAI-2K)^19^ and the SLEDAI renal score (SLEDAI-R).^20^ The GFR was calculated from the serum creatinine concentration using the Schwartz formula.^21^

### Plasma and peripheral blood mononuclear isolation

**Figure 1:**
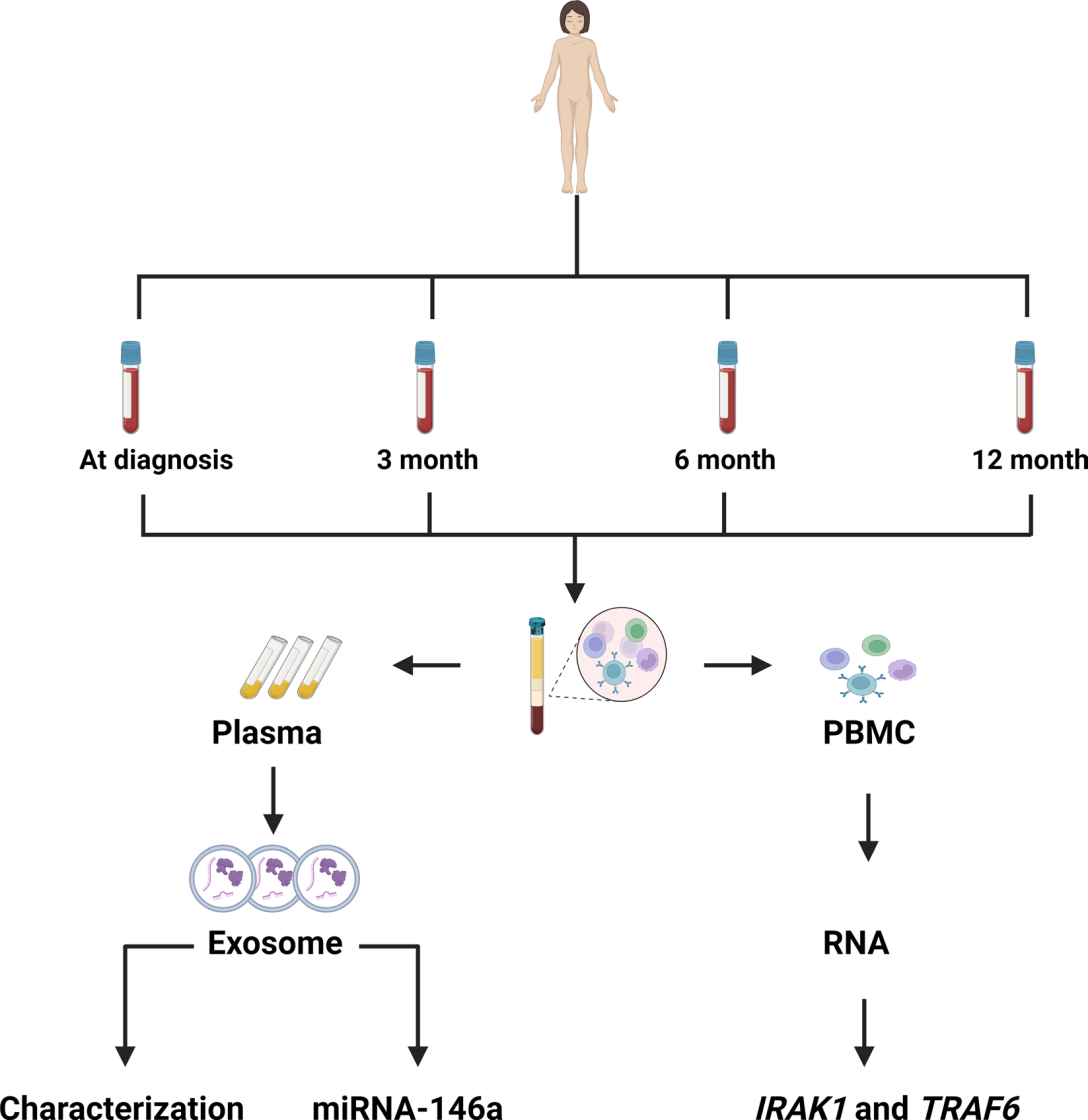
Workflow of this research. Plasma and PBMCs were collected at diagnosis and at 3, 6 and 12 months. *IRAK1*, interleukin-1 receptor-associated kinase 1; miRNA-146a, microRNA-146a; PBMC, peripheral blood mononuclear cell; *TRAF6*, tumour necrosis factor receptor-associated factor 6.

We collected the EDTA blood samples at 0, 3, 6 and 12 months. Figure 1 shows the flow diagram of the laboratory methods. In brief, the blood samples were centrifuged at 1500 rpm for 10 min and separated into plasma and peripheral blood mononuclear cells (PBMCs). The plasma was centrifuged at 3000×g for 15 min and kept at −80°C for exosome isolation, characterisation and evaluation of miRNA-146a expression. PBMCs were used for RNA extraction and evaluation of *IRAK1* and *TRAF6* expression. For PBMC isolation, the buffy coat was collected in a 15 mL tube, and 5 mL of Ficoll-Paque PREMIUM sterile solution density (Cytiva) was added six times following inversion. The tube was centrifuged at 900×g for 30 min. PBMCs were collected, washed with 1× phosphate-buffered saline (PBS) for three times, and kept at −80°C until further use for RNA extraction and evaluation of *IRAK1* and *TRAF6* expression (figure 1).

### Exosome isolation

The plasma samples were pretreated with 4 µL of thrombin at room temperature for 5 min. The plasma samples were subsequently centrifuged at 10 000×g for 5 min, after which a clear portion of the plasma was transferred to a new tube. To eliminate RNA outside the exosome, 0.5 mg/mL of RNase A (Fisher Scientific) was added and the plasma samples were incubated at 37°C for 15 min. Exosomes were isolated using commercial kits (ExoQuick; SystemBiosciences; Palo Alto, California, USA) according to the manufacturer’s instructions.

### Determination of the size and concentration of the exosomes by nanoparticle tracking analysis

Five microlitres of each exosome suspension was diluted with 1 mL of free-particle PBS and was examined using a NanoSight NS300 (NanoSight, Malvern Instrument). A 30 s video was taken with a frame rate of 30 frames/s, and particle movement was analysed using nanoparticle tracking analysis (NTA) V.3.1 software.

### Determination of CD63 expression using western blot analysis

CD63 is a tetraspanin protein that is ubiquitously expressed on exosomes. In this study, the presence of exosomes in the plasma isolates was confirmed using western blot analysis.^22–24^ In brief, 20 µL of exosomes were lysed with 2% sodium dodecyl sulfate (SDS), separated via SDS-polyacrylamide gel electrophoresis at 100 V for 1 hour and subsequently transferred onto nitrocellulose membranes (Bio-Rad). After blocking with 10 mL of Odyssey blocking solution for 1 hour at room temperature, the membranes were incubated with an anti-CD63 antibody. The corresponding secondary antibodies were probed after washing the membranes. The final results were acquired using an Odyssey system (GE) with enhanced fluorescence.

### Transmission electron microscopy

Isolated exosomes (5 µL) were dried on fresh ‘glow-discharged’ 300 mesh formvar/carbon-coated transmission electron microscope (TEM) grids (Ted Pella, Redding, California, USA), negatively stained with 2% aqueous uracil acetate and observed under a Hitachi H7600 TEM (Hitachi High-Technologies Corp, Tokyo, Japan) operated at 50 kV. Images were captured with a side-mounted 1K AMT Advantage digital camera (Advanced Microscopy Techniques, Corp, Woburn, Massachusetts, USA).

### miRNA-146a quantification by using real-time PCR

miRNA isolation was performed using the Serum/Blood Kit (Qiagen, Hilden, Germany) following the manufacturer’s instructions. Briefly, 250 µL of resuspended exosomes were processed with miRNA isolation columns and buffers provided by the manufacturer. A final volume of 30 µL of miRNA solution was collected from each sample using the supplied elution buffer. The miRNA was reverse-transcribed using TaqMan miRNA assays, which were used to perform quantitative real-time PCR (TaqMan Universal Master Mix II, no UNG, Applied Biosystems) for miRNA-146a (assay ID: 002245). In brief, a total of 15 µL of reaction mixture was combined with 10 µL of total miRNA; reverse transcription mixture and primers were incubated at 16°C for 30 min, 42°C for 60 min and 85°C for 5 min. The expression levels of the miRNAs were normalised to those of cel-miR-39 by the delta-delta cycle threshold (DDCt) method using the following formula: DDCt=Ct[miRNA-146a] at 3, 6 and 12 months−Ct[miRNA-146a] at 0 months.

### RNA extraction and reverse transcription

RNA was isolated from PBMCs using the RNeasy Mini Kit (Qiagen) following the manufacturers’s protocols. Briefly, PBMCs isolated as described above were lysed with 150 µL of RLT buffer/beta-mercapto and vortexed for 1 min, after which 1 volume of 70% ethanol was added. The solution was mixed by pipetting up and down three times. After the samples were aspirated, the clear-phase solution was loaded onto a mini-column, and the total RNA was eluted in 30 µL of nuclease-free water. The quantity of isolated RNA was subsequently determined via spectrophotometry using an ND-1000 NanoDrop (Thermo Fisher Scientific). The TaqMan Reverse Transcription Kit (Applied Biosystems, Carlsbad, California, USA) was used to prepare cDNA from 250 ng of total RNA following the manufacturer’s instructions.

### Relative quantification of *IRAK1* and *TRAF6* level by real-time PCR

The gene expression of *IRAK1* and *TRAF6* was measured by using an SYBR Green-based detection technique with an ABI Prism 7500 Real-Time PCR system (Applied Biosystems, Branchburg, New Jersey, USA). Then, cDNA, 6 µL of DNase-free water and 1 µL of specific primers were added (Applied Biosystems, Branchburg, New Jersey, USA). The PCR run was carried out using the thermal profile 50°C for 2 min, 95°C for 10 min, 40 cycles of 95°C for 15 s and 60°C for 1 min on the Rotor gene (Applied Biosystems, Branchburg, New Jersey, USA). The primers used for the genes are shown in table 1. 18S was used as an internal control to normalise the expression of each gene. The relative expression of each gene in every group was calculated using the 2^–ΔΔ^Ct method.

**Table 1:** The sequences of the primers

Primer name		Sequence
*IRAK1*	Forward	GTGCTAGAGACCTTGGCTG
	Reverse	TGTGCTCTGGGTGCTTCTC
*TRAF6*	Forward	ATGCGGCCATAGGTTCTGC
	Reverse	TCCTCAAGATGTCTCAGTTCCAT

*IRAK1*, interleukin-1 receptor-associated kinase 1; *TRAF6*, tumour necrosis factor receptor-associated factor 6.

### Statistical analysis

The data were analysed using Prism V.9 software (GraphPad Software, San Diego, California, USA). Clinical data are presented as the mean±SEM. Repeated measures one-way analysis of variance was performed to detect significant differences in the data among the time points, followed by Dunn’s multiple comparison test. A p value of <0.05 was considered to indicate statistical significance. The Spearman’s correlation coefficient was used to determine the correlation between the circulating exosomal miRNA-146a expression and the levels of *IRAK1* and *TRAF6* mRNA expression, as well as between the circulating exosomal miRNA-146a expression and the clinical data of patients.

## Results

### Patient characteristics

Table 2 summarises the clinical characteristics and laboratory data of the participants at diagnosis and at subsequent follow-ups. There were 12 participants (9 females, 75%) with a mean age at diagnosis of 12.60±0.43 years at initial diagnosis. All the study patients had proliferative lupus nephritis. Six patients (50%) had focal proliferative lupus nephritis (class III) and the other six patients (50%) had diffuse proliferative lupus nephritis (class IV). The histological activity and chronicity indices were 3.4±3.0 and 0.9±0.9, respectively. All participants received prednisolone and hydroxychloroquine. For patients with proliferative lupus nephritis, our centre followed the standard-dose scheme for the dosing and tapering regimen of prednisolone with treatment duration of at least 2 years.^18^ The daily dose of prednisolone at each time point is summarised in table 2. Four patients (33.3%) received induction treatment with a standard dose of intravenous cyclophosphamide 500 mg/m^2^ monthly for 6 months followed by a quarterly dose for 2 years. Eight patients (66.7%) received mycophenolate mofetil at 900–1200 mg/m^2^/day with a maximum dose of 2 g/day for induction and maintenance treatment. The SLEDAI-2K score, SLEDAI-R score, serum albumin concentration, urine protein–creatinine ratio and daily prednisolone dose at subsequent follow-up were significantly different from the levels at diagnosis. All subjects achieved complete remission at 12 months after diagnosis.

**Table 2:** Clinical characteristics and laboratory data of the participants at diagnosis and at subsequent follow-ups

**Parameter**	**Patients with SLE (mean±SEM)**				**P value**		
	**0 months** **(before treatment)**	**3 months**	**6 months**	**12 months**	**0 vs 3 months**	**0 vs 6 months**	**0 vs 12 months**
Age (years)	12.60±0.43	12.79±0.44	13.17±0.42	13.94±0.42	—	—	—
SLEDAI-2K	14.7±1.9	7.0±1.0	3.7±0.5	3.2±0.7	0.0004	0.0001	0.0001
SLEDAI-R	7.7±3.2	4.7±3.3	2.3±2.1	0.7±1.6	0.0015	<0.0001	<0.0001
C3 (mg/dL)	53.08±9.521	105.2±7.185	116.0±8.550	110.1±10.71	<0.0001	0.0004	0.0015
C4 (mg/dL)	6.455±2.051	18.00±2.486	20.25±2.846	20.70±2.737	0.0078	0.0138	0.0007
Serum creatinine (mg/dL)	1.13±0.48	0.63±0.05	0.55±0.04	0.59±0.03	0.157	0.123	0.141
ESR (mm/hour)	55.00±13.15	23.60±3.36	18.63±4.15	12.40±3.98	0.161	0.022	0.098
eGFR (mL/min/1.73 m^2^)	97.83±12.02	107.3±7.49	121.8±9.98	115.3±6.52	0.202	0.052	0.086
Albumin (g/dL)	3.13±0.20	3.66±0.16	4.01±0.06	4.26±0.06	0.028	0.0004	0.0001
UPCR (mg/mg)	2.17±0.39	1.42±0.34	0.75±0.28	0.17±0.11	0.015	0.002	0.0001
WBCs	13010±2230	12203±1232	9344±809.10	7677±676.80	0.379	0.082	0.016
Hb (g/L)	98.3±4.8	115.8±4.9	126.0±3.5	110.8±7.3	0.014	0.0003	0.073
Platelet (×10^9^/L)	35033±35187	362417±26899	350250±25608	350583±24476	0.304	0.499	0.497
Prednisolone dose (mg/day)	0	35.8±16.6	16.7±10.7	7.2±5.8	0.0054	< 0.0001	<0.0001

eGFR, estimated glomerular filtration rate; ESR, erythrocyte sedimentation rate; Hb, haemoglobin; SLEDAI-2K, SLE Disease Activity Index 2000; SLEDAI-R, SLEDAI renal score; UPCR, urine protein/creatinine ratio; WBCs, white blood cells.

### The size and concentration of exosomes in JPLN

**Figure 2:**
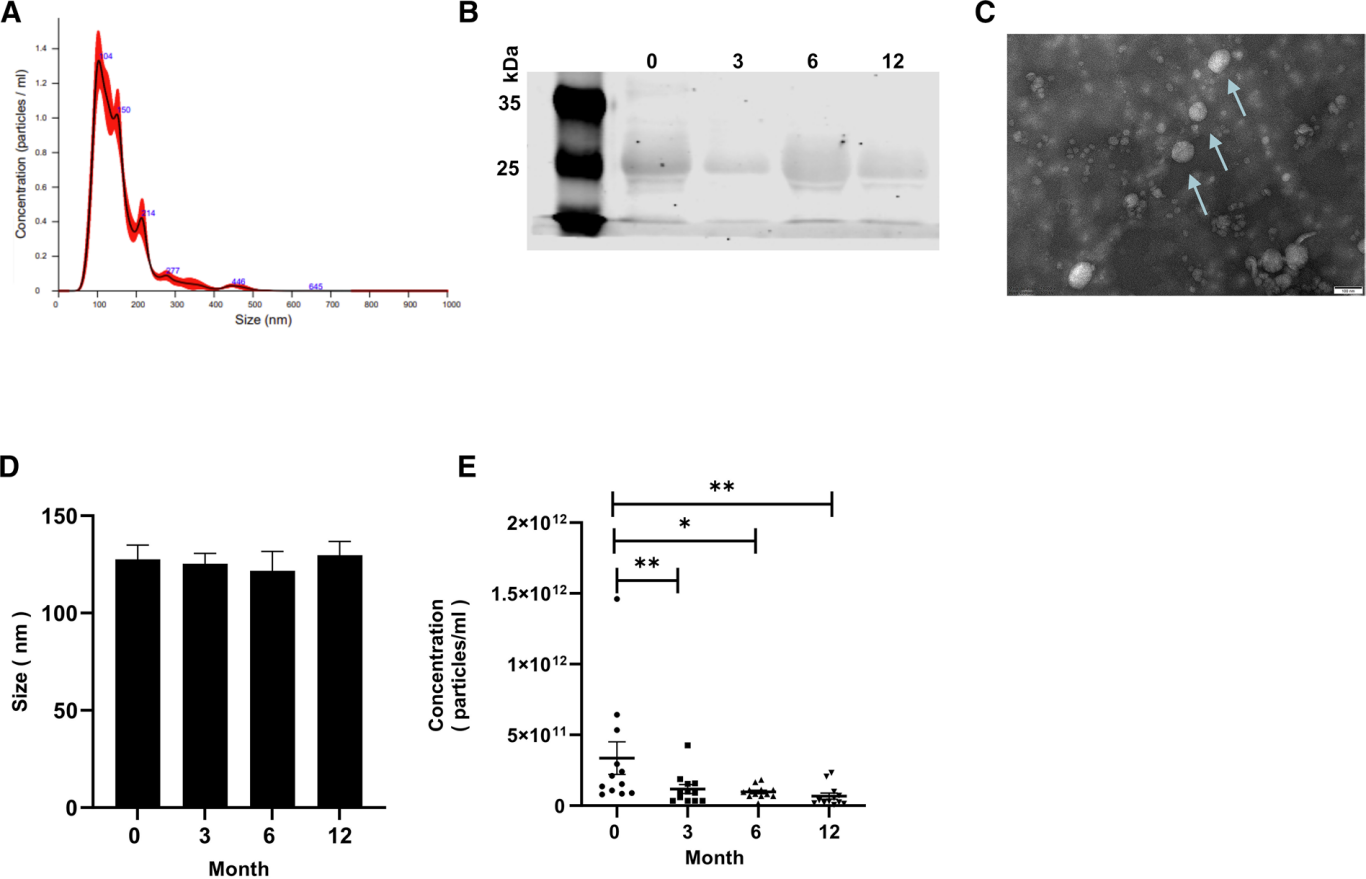
Characterisation of exosomes isolated from human plasma. (A) Representative NTA image showing the size of the x-aixs and the concentration of exosomes on the y-axis. (B) The expression of exosome marker CD63 was determined by western blotting. The lanes from left to right are 0, 3, 6 and 12 months. (C) Transmission electron microscopy image of exosomes, bar=100 nm. (D) Representative size range of exosomes in the groups. (E) Representative concentrations of exosomes in the different groups. The data were compared among time points using repeated measures one-way analysis of variance. *P=0.0043; 0 vs 6 months, **p=0.0012; 0 vs 3 months and 0 vs 12 months. NTA, nanoparticle tracking analysis.

The exosomes were characterised using NTA (size and concentration, figure 2A), western blot analysis of CD63 (a marker of exosome, figure 2B) and TEM (morphology of the exosome, figure 2C). The exosomes were visualised via TEM as round particle with diameters ranging from 100 to 150 nm via NTA. Moreover, there was no significant change in the size of the exosomes in patients with JPLN at different time points (figure 2D), but the concentrations of the exosomes were significantly decreased at subsequent follow-ups (figure 2E).

### The expression of circulating exosomal miRNA-146a in JPLN and its association with the levels of *IRAK1* and *TRAF6* mRNA

**Figure 3:**
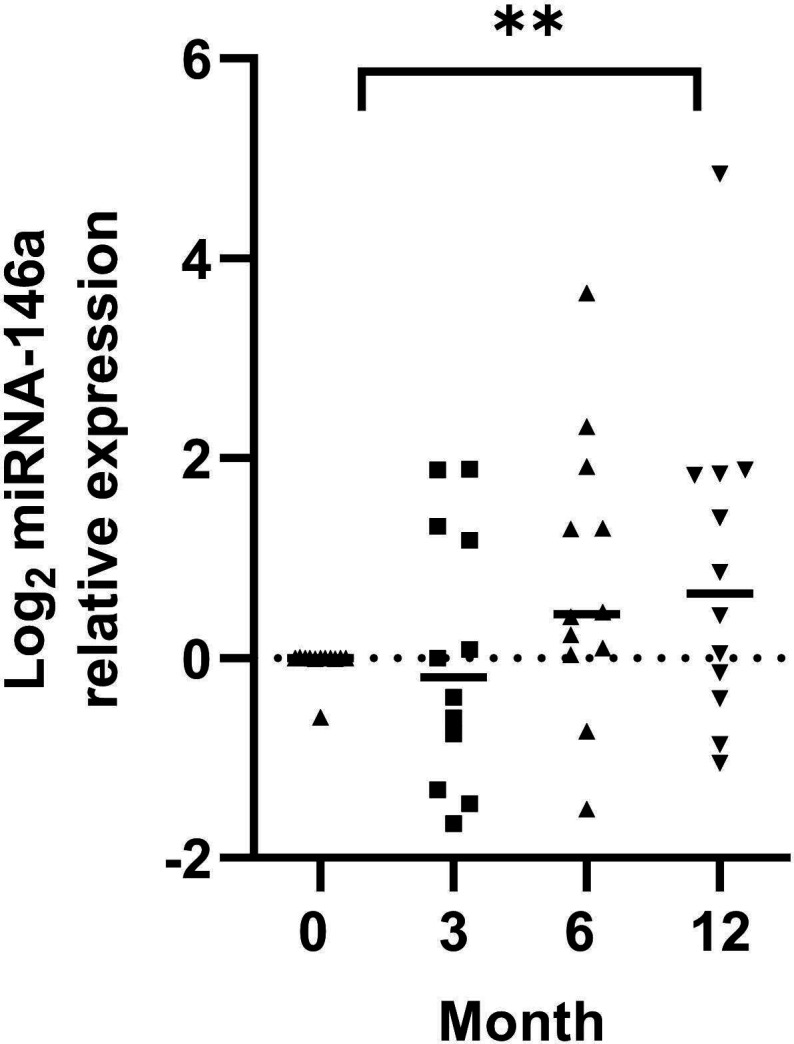
Circulating exosomal miRNA-146a expression in longitudinal samples. **P<0.001. miRNA-146a, microRNA-146a.

**Figure 4:**
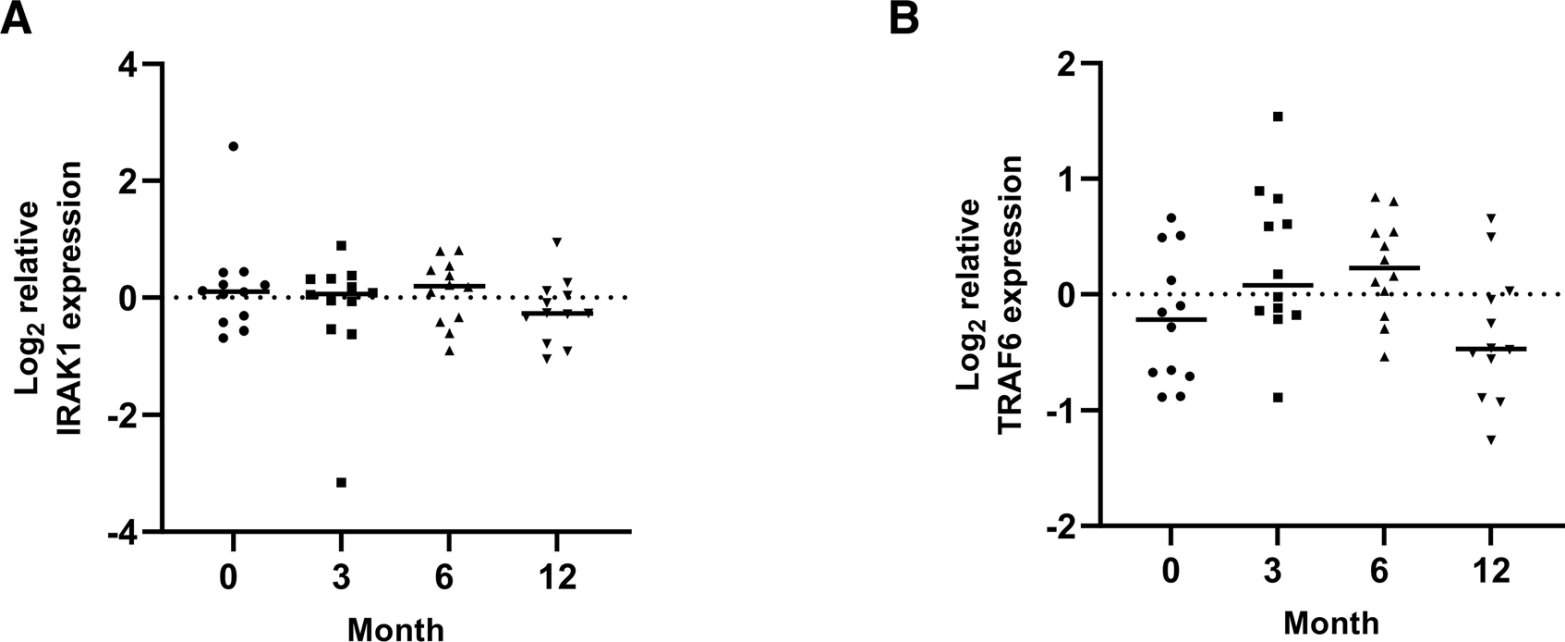
The expression of *IRAK1* (A) and *TRAF6* mRNA (B) in longitudinal samples. *IRAK1*, interleukin-1 receptor-associated kinase 1; *TRAF6*, tumour necrosis factor receptor-associated factor 6.

**Figure 5:**
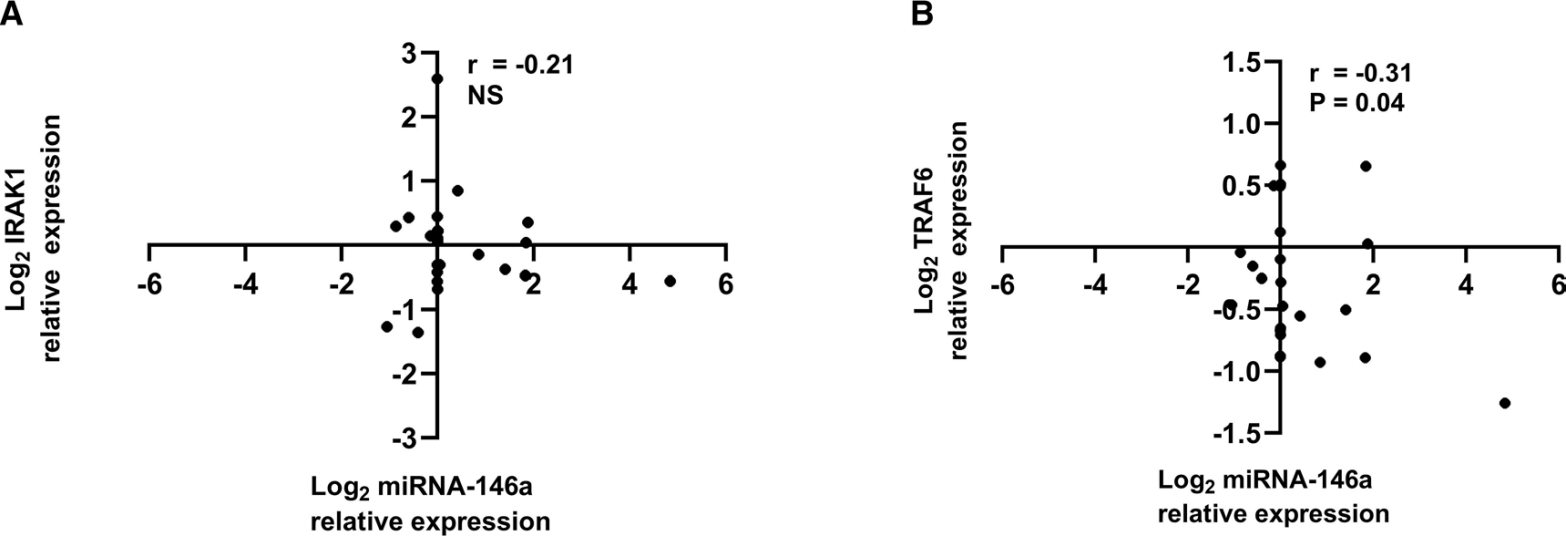
The correlation between exosomal miRNA-146a expression and the expression of the target genes: *IRAK1* (A) and *TRAF6* (B). The data were compared by Spearman’s rank correlation coefficient. *IRAK1*, interleukin-1 receptor-associated kinase 1; miRNA-146a, microRNA-146a; NS, not significant; *TRAF6*, tumour necrosis factor receptor-associated factor 6.

The expression levels of circulating exosomal miRNA-146a were similar at diagnosis and at 3 and 6 months. However, there was a significant increase in the level of circulating exosomal miRNA-146a at 12 months compared with that at diagnosis (figure 3). Although the *IRAK1* and *TRAF6* mRNA levels did not significantly differ at different time points, there was a trend toward a decrease in the levels of both genes at 12 months (figure 4A,B). There was a negative correlation between circulating exosomal miRNA-146a expression and the level of *TRAF6* mRNA (r=−0.30, p=0.04, figure 5B). We did not find an association between the circulating exosomal miRNA-146a expression and the level of *IRAK1* mRNA (r=−0.21, p=NS, figure 5A).

### Correlation of miRNA-146a with clinical parameters of lupus nephritis

**Figure 6:**
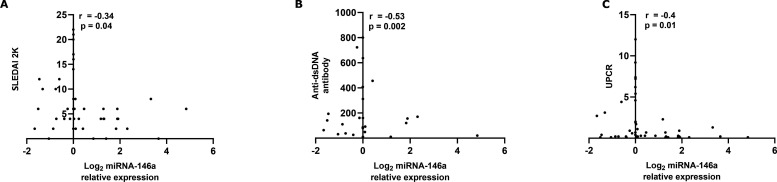
Correlations between exosomal miRNA-146a and the (A) SLE Disease Activity Index 2000 (SLEDAI-2K), (B) anti-dsDNA antibody and (C) UPCR. The data were compared by Spearman’s rank correlation coefficient. anti-dsDNA, anti-double-stranded DNA; miRNA-146a, microRNA-146a; UPCR, urine protein–creatinine ratio.

Next, we investigated the correlation between miRNA-146a and various clinical parameters in juvenile patients with SLE. Circulating exosomal miRNA-146a expression was significantly inversely correlated with SLEDAI-2K (r=−0.34, p=0.04), anti-double-stranded DNA (anti-dsDNA) antibody (r=−0.53, p=0.002) and urine protein–creatinine ratio (r=−0.40, p=0.01), as shown in figure 6A–C, respectively.

## Discussion

The presence of lupus nephritis can significantly affect the overall disease course and prognosis of patients with SLE. Patients with JSLE frequently necessitate elevated doses and extended duration of immunosuppressive therapy compared with those with adult-onset SLE, thereby increasing the potential for drug-related adverse effects.^4 5^ Therefore, investigating the monitoring of disease activity and treatment response in patients with JPLN is important. miRNA-146a is actively involved in the pathogenesis of lupus nephritis and might be transferred throughout the body via extracellular vesicles.^25^ Exosomes are tiny extracellular vesicles with a double-layer membrane resembling the cell membrane that various cell types secrete to transfer various molecules, including miRNAs, to other cells.^26^ The present study showed the correlation between the expression levels of exosomal miRNA-146a and its possible consequential impact on downstream targets, including *IRAK1* and *TRAF6*, within the context of JPLN, a non-invasive approach for monitoring treatment in patients with JSLE.

In the present study, we found that the concentration of exosomes was significantly decreased at 3, 6 and 12 months in correlation with good treatment outcomes similar to the findings of a previous report.^27^ A higher exosome abundance is detected in patients with lupus which is correlated with SLE disease activity, as is a low exosome abundance in healthy controls.^27^ Moreover, we also found that the level of circulating exosomal miRNA-146a was higher at 12 months after successful treatment. Our results agree with Wang *et al*, who showed that the upregulation of miRNA-146a expression was associated with treatment.^28^ Tang *et al* showed that miRNA-146a was increased in the T cells of patients with lupus nephritis treated with mycophenolic acid compared with those who were untreated.^9^ We found that the correlations between the upregulation of circulating exosomal miRNA-146a and the SLEDAI-2K score, anti-dsDNA antibody level and proteinuria were in agreement with the findings of Wang *et al*.^28^ Hence, miRNA-146a might be correlated with lupus activity due to its anti-inflammatory property and the detection of miRNA-146a in exosomes, a protective mechanism of miRNAs from the circulatory microenvironment, might be useful for determining lupus activity. The development of SLE symptoms is possibly linked to a reduction in miRNA-146a levels which disrupts the regulation of the interferon (IFN) pathway.^29^ miRNA-146a expression can regulate IFN production which is mediated by *IRAK1* and *TRAF6*, two key elements involved in Toll-like receptor signalling.^29^ The results of the present study indicated that the expression of *IRAK1* and *TRAF6* showed a tendency to be downregulated at 12 months compared with that at diagnosis, and that there was a significant negative correlation between circulating exosomal miRNA-146a and *TRAF6*. We observed an individual who exhibited a very high circulating miRNA-146a expression. Notably, this patient showed a markedly favourable response to treatment and achieved complete remission in a relatively shorter time than did the other patients. At 12 months, the expression of miRNA-146a in this patient was extremely high. This result is similar to that of Zheng *et al*^29^ who reported that the upregulation of miRNA-146a and downregulation of *TRAF6* can significantly inhibit the *NF-κB* transcriptional activity of glomerular mesangial cells. Similarly, Taganov *et al*^30^ reported that the overexpression of miRNA-146a inhibits the activity of luciferase reporter plasmids containing either the 3’ untranslated regions of *IRAK1* or *TRAF6* in the human embryonic kidney cells. However, in this study, we did not find a correlation between circulating exosomal miRNA-146a and *IRAK1*. One potential explanation for this difference in expression patterns was the presence of circulating miRNA-146a target genes, such as *IRAK1*, in patients with JSLE. These differences may hinder the ability of miRNA-146a to accurately recognise its own set of target genes and the lack of complete concerted downregulation of NF-κB upstream molecules, in the presence of increased miRNA-146a expression.

There are some limitations to the study. Selection of immunosuppressive regimen was based on physician discretion in our centre. Difference in classification of lupus nephritis, histological activity and chronicity indices, prednisolone dosing, immunosuppressive regimen and response to treatment could be important confounding factors and may alter the expression of miRNA-146a and proinflammatory pathways.

In summary, this study was the first to report that circulating exosomal miRNA-146a in patients with JSLE might inhibit lupus-induced inflammation through *TRAF6* downregulation. The potential use of circulating exosomal miRNA-146a might be a non-invasive biomarker for lupus nephritis.

## Data Availability

Data are available upon reasonable request. The datasets generated during and/or analysed during the current study are available from the corresponding author on reasonable request.
